# Inequalities in general practice remote consultations: a systematic review

**DOI:** 10.3399/BJGPO.2021.0040

**Published:** 2021-04-28

**Authors:** Ruth F Parker, Emma L Figures, Charlotte AM Paddison, James IDM Matheson, David N Blane, John A Ford

**Affiliations:** 1 Medical Student, University of Cambridge, Cambridge, UK; 2 GPST2 Registrar, West Cambridgeshire GP Training Programme, Cambridge, UK; 3 Senior Fellow, Nuffield Trust, London, UK; 4 Chair, Health Inequalities Standing Group, Royal College of General Practitioners, London, UK; 5 Clinical Research Fellow in General Practice and Primary Care, Institute of Health and Wellbeing, University of Glasgow, Glasgow, UK; 6 Clinical Lecturer in Public Health, Department of Public Health and Primary Care, University of Cambridge, Cambridge, UK

**Keywords:** Primary Health Care, Telemedicine, Socioeconomic Factors, Systematic Review

## Abstract

**Background:**

COVID-19 has led to rapid and widespread use of remote consultations in general practice, but the health inequalities impact remains unknown.

**Aim:**

To explore the impact of remote consultations in general practice, compared to face-to-face consultations, on utilisation and clinical outcomes across socioeconomic and disadvantaged groups.

**Design & setting:**

Systematic review.

**Method:**

The authors undertook an electronic search of MEDLINE, EMBASE, and Web of Science from inception to June 2020. The study included studies that compared remote consultations to face-to-face consultations in primary care and reported outcomes by PROGRESS Plus criteria. Risk of bias was assessed using ROBINS-I. Data were synthesised narratively.

**Results:**

Based on 13 studies that explored telephone and internet-based consultations, this review found that telephone consultations were used by younger people of working age, the very old, and non-immigrants, with internet-based consultations more likely to be used by younger people. Women consistently used more remote forms of consulting than men. Socioeconomic and ethnicity findings were mixed, with weak evidence that patients from more affluent areas were more likely to use internet-based communication. Remote consultations appeared to help patients with opioid dependence remain engaged with primary care. No studies reported on the impact on quality of care or clinical outcomes.

**Conclusion:**

Remote consultations in general practice are likely to be used more by younger, working people, non-immigrants, older patients, and women, with internet-based consultations more by younger, affluent, and educated groups. Widespread use of remote consultations should be treated with caution until the inequalities impact on clinical outcomes and quality of care is known.

## How this fits in

General practice has undergone a rapid transformation from face-to-face to remote consultations, but the impact on inequalities remains unknown. This is the first review to look at the impact of remote consultations on health inequalities. This study found that remote consultations are likely to be used more by younger working people, non-immigrants, older patients, and women, with internet-based consultations more by younger, affluent, and educated groups. However, no studies reported the impact on clinical outcomes or quality of care.

## Introduction

Remote consultations in general practice have become increasingly important over the past year. GPs in the UK have been advised to use remote consultations, wherever possible, to protect patients and staff from COVID-19.^1^ In April 2020, NHS England and NHS Improvement published guidance on establishing a remote ‘total triage’ model in general practice using online consultations.^2^ Recent data confirmed marked shifts in consultation patterns, from roughly 90% of appointments being face-to-face before COVID-19 to mostly remote consulting during COVID-19.^3^ However, little is known about the impact on access, utilisation, safety, quality, and outcomes of remote consultations.

Remote consulting was used before COVID-19, offering potential benefits in access, especially for those in rural areas or with mobility issues, and appointment flexibility. It is potentially more cost- and time-effective than face-to-face consultations, and has been explored as a possible solution to increased GP workload and GP shortages.^4^ Remote consulting includes telephone and video consultations, as well as synchronous or asynchronous internet-based communications, such as emails or web-based consultation platforms.

However, previous research has highlighted the risk of remote consultation widening health inequalities.^5^ For example, remote consulting may create barriers for those with limited or no internet access, those who do not speak English, and people with learning difficulties or mental illness. As general practice continues to evolve, up-to-date evidence is needed to understand the implications of remote consultations for those most in need, who are often least able to access help.^6^ The pre-pandemic patterns of patient access and engagement with remote consultations can inform the field's understanding of whether disadvantaged patients are likely to benefit or not during the widespread change in delivery of primary care. Therefore, the aim of this systematic review was to explore the impact of remote consultations in general practice, compared to face-to-face consultations, on utilisation and clinical outcomes within and between socioeconomic and disadvantaged groups.

## Method

The study undertook a systematic review using Cochrane methodology to explore inequalities in remote consultations in general practice. The review was registered on PROSPERO and guided by a protocol.

The authors took a broad definition of remote consultations in general practice, or equivalent primary care organisations, including telephone, video, or web-based consultations, which could be synchronous or asynchronous. The authors conceptualised inequalities using the PROGRESS Plus criteria^7^ and NHS England categories of inclusion health groups.^8^ PROGRESS refers to place of residence, race/ethnicity/culture/language, occupation, gender/sex, religion, education, socioeconomic status, and social capital. It also refers to personal characteristics associated with discrimination (for example age, or disability), features of relationships, and time-dependent relationships. NHS inclusion health groups include those experiencing homelessness, vulnerable migrants, sex workers, and those from the Gypsy, Roma, and Traveller communities. The study's main outcomes were utilisation or clinical outcomes. Importantly, the comparison was with face-to-face consultations to provide a suitable counterfactual.

The authors undertook an electronic search of MEDLINE, EMBASE, and Web of Science from inception to June 2020 (MEDLINE search strategy shown in supplementary materials). The search strategy combined three broad categories of terms: 1) health inequalities terms as developed by Prady and colleagues;^9^ 2) primary care terms as developed by Gill and colleagues;^10^ and 3) telemedicine terms used by Downes and colleagues.^11^ The authors also searched the references of complementary reviews, and undertook a grey literature search of targeted websites (for example Health Foundation, King’s Fund, Nuffield Trust) and a broader internet search.

All titles and abstracts were screened according to eligibility criteria (see Box 1) by two researchers, and a third senior researcher quality checked 20%. Articles were categorised as included, excluded, or unclear. All unclear titles were reviewed, and any disagreements were resolved through discussion. The full text of all included or unclear articles was identified and screened for eligibility.

Box 1 **Inclusion and exclusion criteria**

Inclusion criteria

- Any study design that presented differences between socioeconomic or disadvantaged groups. This may include randomised controlled trials, before and after studies, comparative observational studies, or non-randomised studies.
- The study must include a remote consultation and a comparison or face-to-face consultations.
- The study must present results by PROGRESS Plus criteria or NHS England categories.

Exclusion criteria

- Studies that were not undertaken in primary care.
- Studies without a suitable comparator.
- No exclusions based on language.

Data extracted from the articles included aim, study design, data sources, population, intervention, length of study, and results by PROGRESS Plus group. Risk of bias was assessed using the Cochrane ROBINS-I tool.^12^ Data extraction and risk of bias assessments were conducted by one author and checked by a senior researcher, with discrepancies resolved through discussion. Results were synthesised narratively. Meta-analysis was not undertaken due to methodological heterogeneity.

## Results

### Study characteristics and risk of bias

The database search identified 8969 studies, with 6645 after de-duplication (Supplementary Figure S1). Grey literature searching identified 87 further studies. A total of 13 met the eligibility criteria.^5,13–24^ Study design included eight retrospective longitudinal studies, three cross-sectional surveys, one interrupted time series, and one mixed-methods study (Supplementary Table S1).

Data was sourced primarily from electronic medical records, insurance claims, and surveys. The number of participants varied from 1058^17^ to 2.4 million,^21^ and only three studies had fewer than 5000 participants.^17,19,20^ Study length ranged from 1 week^17^ to 14.3 years.^20^ Four of the studies were from the USA,^13,14,16,20^ two from the UK,^5,19^ two from Denmark,^17,24^ and one each from Canada,^18^ Italy,^15^ Sweden,^23^ Spain,^21^ and The Netherlands.^22^ All but two studies^13,24^ were published in the last decade.

Six studies reported the use of telephone, email, or video-calls compared to traditional office-based consultations.^5,13,17,21–23^ Four studies focused on specific interventions (Teladoc telephone or video consults, booked online;^16^ Telephone First, in which patients have an initial phone consultation, followed by face-to-face if necessary;^19^ eVisits, an online questionnaire;^14^ and an inner-city telemedicine service.^20^ Two studies focused on remote consultation use by migrants,^15,24^ and one on patients with opioid dependence.^18^

Age and gender were the commonest reported PROGRESS Plus criteria (*n* = 8 and *n* = 7, respectively), followed by socioeconomic status (*n* = 5), and place of residence (*n* = 4) (Supplementary Table S2). No studies reported results by religion or social capital.

All studies reported primary care utilisation, but none reported clinical or quality of care outcomes.

Risk of bias was high in five studies,^5,13,15,17,22^ and moderate in eight.^14,18–21,23,24^ Eight of the included studies had a high risk of bias due to confounding.^5,15,17,18,21–24^ Two studies had a moderate risk of bias due to missing data.^5,13^ The rest had a low risk or no information (Supplementary Table S3).

### Inequalities in remote consultations

#### Age

Eight studies reported results by age.^5,13,14,16,17,21–23^ Atherton *et al^5^* and Gonzalez *e* *t al^21^* found that telephone consultations were more likely in the very old (over 85 years) and least likely for those under 5 years old. Atherton *et al* also found higher telephone consultation use in 25 to 44 year olds, whereas Gonzalez *e* *t al* did not.^5,21^ Six studies found that digital or internet-based consultations were more likely to be used by younger patients.^5,13,14,16,17,23^ Huygens *e* *t al* reported mean age for face-to-face, telephone, and e-mail with large standard deviations, making the distinction between age groups difficult.^22^

#### Gender

Seven articles reported results by gender.^5,13,14,16,17,21,22^ In all of these, women were more likely to use remote consultations compared to face-to-face consultations than men, although the difference was small. Gonzalez *e* *t al* ^21^ found that women in Spain were statistically significantly more likely to use telephone consultations than face-to-face consultations (2.0–2.3% difference between men and women).^21^ Similarly, Atherton *et al* found a 2% difference between men and women in the proportion of all consultations that were telephone-based (17% versus 19%).^5^ Uscher-Pines and Mehrotra found that women were more likely to use internet-based remote consultation (5.9% of all consultations in women compared to 4.9% in men),^16^ and Beckjord *et al*'s study of internet-users in 2005 found that women were more likely to have communicated online with healthcare providers compared to men (odds ratio 1.47; 95% confidence interval = 1.00 to 2.15).^13^

#### Socioeconomic factors

Nine studies reported results by a measure of deprivation, income, education, or occupation.^5,13,14,16,17,19,20,22,23^ Three studies found no difference between groups; Newbould *et al* found no difference between employed and unemployed in the use of Telephone First;^19^ Uscher-Pines and Mehrotra found no income gradient in Teladoc use;^16^ and Beckjord *et al* found no difference in the educational qualifications of people who had communicated with their healthcare provider via the internet compared to those who had not.^13^

However, Huygens *et al* found that more affluent patients in The Netherlands were more likely to use email compared to telephone or face-to-face consultations.^22^ Furthermore, Ekman *et al* found that patients in affluent areas of Sweden were more likely to use digital consultations,^23^ and Bertelsen and Stub Petersen found that people with higher educational backgrounds were more likely to report having used technology to communicate with their GP (72%, compared with 46% of those with no professional education).^17^ Mehrotra *et al* found no income gradient in the use of eVisits, but employed people were more likely to use the service.^14^ Atherton *et al* reported a higher proportion of telephone consultations compared to face-face in patients living in relatively deprived areas (21.6% telephone consultations in most deprived quintile compared to 16.4% in least deprived, unadjusted for age or sex).^5^ Ronis *et al* found the addition of a telemedicine service to usual care increased access to acute illness visits for inner city children compared to suburban children.^20^

#### Ethnicity and immigrants

Three studies report results by ethnicity.^5,13,14^ Atherton *et al* found that people from non-White ethnicities had higher unadjusted telephone consultation use as a proportion of all consultations, compared to White people (21.2% compared with 18.1%).^5^ However, Mehrotra *et al* found that White people had a higher proportion of eVisits compared to Black or African American people (7.5% compared with 3.1%).^14^ Furthermore, Beckjord *et al* found mixed results across White, Hispanic, African American, and Asian American people in online communication with healthcare providers.^13^ Two studies looked at immigrants, in Denmark and Italy. Both found that native residents had higher telephone consultation use as a proportion of all consultations compared to immigrants.^15,24^

#### Place

All three studies considering place found urban areas were more likely to have higher telephone consultations and use of digital methods to contact primary care. However, none of these results were adjusted for potential confounders such as practice innovativeness, raising the possibility that this pattern reflects the type of practices that operate in urban areas.^13,21,23^

#### Disadvantaged groups

One study looked specifically at patients with opioid addictions and found that remote consultations improved engagement with primary care compared to face-to-face. This study, which included over 5000 patients, found that 59% of opioid users remained engaged with telephone appointments, compared to 48% with face-to-face.^18^

## Discussion

### Summary

The authors identified 13 studies comparing remote consultations in primary care to face-to-face consultations.^5,13–24^ All had moderate or high risk of bias. This review's findings suggest that telephone consultations are used more by younger working-age people, the very old, and non-immigrants, with internet-based consultations more likely to be used by younger people. Women consistently used more remote consulting than men, though this difference was small. Socioeconomic and ethnicity findings were mixed, with weak evidence that patients from more affluent areas were more likely to use digital consultations. In one study, remote consultations resulted in more patients with opioid dependence remaining engaged with primary care.^18^ None of the studies reported on the impact of remote consultations on quality of care or clinical outcomes.

#### Meaning of the findings

The higher use of telephone consultations among younger people suggested ease of access, convenience, and capability, while for older people this may be an imposition resulting from lower mobility and limited GP resources for visits. Older patients may be more likely to benefit from a face-to-face consultation.^25^ Age patterns may reflect differences between urban and rural areas, with urban areas typically having a younger, more technologically literate population. The sense of community in rural areas may encourage a tendency toward face-to-face consultations.^26^ Improved retention rates by almost a quarter for patients with opioid dependence may be due to the generally younger patient population, or pre-existing relationships with the clinician. The reasons for the small, but consistent, difference between men and women are unknown, but may reflect gender preferences or patterns of disease.^27^ Language barriers, unfamiliarity with new health systems, and genuine or perceived exclusion from care may explain lower use by immigrants. The mixed ethnicity findings were unsurprising because ethnicities were combined in most of the studies, making it impossible to unpick the substantial heterogeneity between and within groups. The greater use of remote consultations among the employed is expected as it is more convenient to employment. More affluent patients may have had greater access to technologies and digital literacy, although less affluent patients may benefit from remote consultations if their employment is uncertain or less flexible (for example, agency work).^28^

Importantly, this review highlights the evidence gaps. While conclusions can be drawn on who is more or less likely to use remote consultations, it is not known what this means for patient experience, safety, secondary or community healthcare use, or clinical outcomes.

### Strengths and limitations

This is the first review to look at the impact of GP remote consultations on inequalities. This study used a robust review methodology, strengthened by the most relevant comparator, and a broad conceptualisation of inequalities. However, the absence of data on clinical outcomes or quality of care means that results were limited to utilisation. Only a few PROGRESS Plus criteria were reported; for example, disability was not included. All studies had a high or moderate risk of bias. It is likely that people with multiple disadvantage — such as someone who belongs to an ethnic minority group *and* on a low income *and* with complex health needs — are at higher risk of inequalities, but no articles considered intersectionality. Studies were heterogeneous in terms of country and population. None of the studies showed the impact of rapid transformation of primary care in the context of a pandemic, so it is unknown whether the findings can be generalised to these circumstances.

### Comparison with existing literature

Mold and colleagues undertook a review of e-consultation techniques, such as secure email, messaging, or video links, in primary care.^29^ Similar to the present study's findings, their review found that younger employed adults were more likely to use digital communication. While these techniques improved patient satisfaction and engagement, the authors also found concerns from health professionals around quality and health outcomes. In keeping with the present study's findings, a separate evaluation of a digital GP service found that people who used digital primary care tended to be younger and more affluent.^30^ In a systematic review of telephone consultations in primary care, Bunn and colleagues found that telephone consultations may reduce GP workload, but could not draw conclusions about the impact on quality or outcomes, and did not report results by socioeconomic or disadvantaged group.^4^ In contrast, Newbould and colleagues found that tele-first increased GP workload by 8%.^19^

Hewitt and colleagues undertook a qualitative study in 2010 to compare telephone and face-to-face consultations.^31^ The authors found telephone consultations were generally shorter and meant that doctors did not identify additional concerns or problems.

### Implications for research and practice

A key research priority is understanding the impact of remote consultations in primary care on clinical outcomes, and on measures of quality of care such as patient-reported experience and relational continuity. Evaluations should explicitly assess the benefits and harms to vulnerable and disadvantaged patients, taking a cumulative risk approach to understand the impact of multiple disadvantage and clinical complexity. Research needs to distinguish between different types of remote and digital care, focusing on what works, when, and for whom.

In the UK, there is a policy directive to make all primary care consultations remote by default; based on current evidence, it is impossible to draw conclusions about the impact of remote consulting on clinical outcomes, or to rule out that it may increase inequalities. A mandatory and rapid shift towards ‘digital first’ primary care may not work well for all patients, as has been highlighted by the Royal College of General Practitioners,^32,33^ and as supported by recent evidence from Scotland.^34^ Making remote consultations the default is premature given the lack of evidence on clinical outcomes and patient experience for vulnerable and disadvantaged patients. Primary care policy needs to maintain flexibility, and sits alongside the commitment to reduce health inequalities in access and outcome. An alternative policy position is that '*video consulting should continue to be offered after physical distancing is over, but it is not universally appropriate. It should therefore be an option rather than mandatory*', as recommended by the NHS Digital Health & Care Directorate in their Equality Impact Assessment of the Near Me Video Consulting Programme.^34^ Greater use of Health Inequalities Impact Assessments and Equity Audits could support and inform policy decisionmaking.

Remote consultations compared to face-to-face consultations in general practice are likely to be used more by younger working people, non-immigrants, and women, with internet-based consultations used more by younger, affluent, and educated groups, and telephone consultations by the very old. The impact of remote consultations on quality and clinical outcomes remains unknown.
